# Lectures on the Principles and Practice of Physic

**Published:** 1859-01

**Authors:** 


ART. VIII.—*Lectures on the Principles and Practice of Physic*; delivered at King's College, London. By THOMAS WATSON, M.D., Fellow of the Royal College of Physicians, etc. etc. A new American, from the last revised and enlarged London edition. With additions, by D. FRANCIS CONDIE, M.D., Fellow of the College of Physicians of Philadelphia. 8vo., pp. 1224. Philadelphia: Blanchard & Lea, 1858.

THE mere announcement of the appearance of a new edition of the elaborate work of Dr. Watson is sufficient to insure it a wide circulation, without our bestowing any praise upon it as a literary production. In our student days we well remember with what pleasure we always took it up, with what delight we read its fascinating pages, and how we admired the clearness, grace, and smoothness of the author's style, and the vast amount of erudition stored up in it. It now reappears much increased in size, and we greet it as we would a long-absent friend. It has been the duty of Dr. Condie to incorporate in the text such facts as had been omitted by Dr. Watson, and to bring it up to the wants of the American student. His additions on cholera infantum, cerebro-spinal meningitis, yellow fever, bilious remittent fever, and on other topics, are interesting, and enhance the value of the work. From its bulky size, we could wish that it had preserved its English form of two volumes, as in this shape it would be much more easy to handle, and, in addition, would have the great advantage of larger type, a great assistance, we need not say, to those who read at night.

